# Psychological Determinants of Consumer Acceptance of Personalised Nutrition in 9 European Countries

**DOI:** 10.1371/journal.pone.0110614

**Published:** 2014-10-21

**Authors:** Rui Poínhos, Ivo A. van der Lans, Audrey Rankin, Arnout R. H. Fischer, Brendan Bunting, Sharron Kuznesof, Barbara Stewart-Knox, Lynn J. Frewer

**Affiliations:** 1 Faculty of Nutrition and Food Sciences, University of Porto, Porto, Portugal; 2 Marketing and Consumer Behaviour Group, Wageningen University, Wageningen, The Netherlands; 3 Northern Ireland Centre for Food and Health, University of Ulster, Coleraine, United Kingdom; 4 School of Psychology, University of Ulster, Londonderry, United Kingdom; 5 Food and Society Group, SAFRD, Newcastle University, Newcastle Upon Tyne, United Kingdom; 6 Division of Psychology, University of Bradford, Bradford, United Kingdom; University of Rome, Italy

## Abstract

**Objective:**

To develop a model of the psychological factors which predict people’s intention to adopt personalised nutrition. Potential determinants of adoption included perceived risk and benefit, perceived self-efficacy, internal locus of control and health commitment.

**Methods:**

A questionnaire, developed from exploratory study data and the existing theoretical literature, and including validated psychological scales was administered to N = 9381 participants from 9 European countries (Germany, Greece, Ireland, Poland, Portugal, Spain, the Netherlands, the UK, and Norway).

**Results:**

Structural equation modelling indicated that the greater participants’ perceived benefits to be associated with personalised nutrition, the more positive their attitudes were towards personalised nutrition, and the greater their intention to adopt it. Higher levels of nutrition self-efficacy were related to more positive attitudes towards, and a greater expressed intention to adopt, personalised nutrition. Other constructs positively impacting attitudes towards personalised nutrition included more positive perceptions of the efficacy of regulatory control to protect consumers (e.g. in relation to personal data protection), higher self-reported internal health locus of control, and health commitment. Although higher perceived risk had a negative relationship with attitude and an inverse relationship with perceived benefit, its effects on attitude and intention to adopt personalised nutrition was less influential than perceived benefit. The model was stable across the different European countries, suggesting that psychological factors determining adoption of personalised nutrition have generic applicability across different European countries.

**Conclusion:**

The results suggest that transparent provision of information about potential benefits, and protection of consumers’ personal data is important for adoption, delivery of public health benefits, and commercialisation of personalised nutrition.

## Introduction

Poor nutrition contributes to the incidence of many diseases, see *inter alia*, [1]–[5]. It has been estimated that approximately 80% of cases of cardiac disease, stroke, type 2 diabetes, and 40% of cancers could be avoided through improved lifestyle, including those related to diet [6]. However, there may be substantial *genetically determined* variation *between* individuals in what constitutes an optimal diet with regard to health protection [7]. *Nutrigenomics* is the study of the effects of foods and food constituents on gene expression and health. Personalised nutrition, or personalised dietary advice, which can also be based on an individual’s genotype, can be translated into personalised dietary recommendations [8]–[9]. The advantage of nutrigenomics-based nutrition advice over and above that based on age, sex, body mass index (BMI), diet, physical activity and health status, is that genetic differences between individuals, which may interact with phenotype and co-determine health impacts of dietary choices, are explicitly taken into account [10]. Various (primarily internet based) personalised nutrition and nutrigenomics based personalised dietary advice services are currently, and increasingly, available commercially [11], although consumer acceptance of nutrigenomics may vary between individuals and is not assured [12]. This is, in part, because some consumers may be concerned about the commercialisation of a technology which utilises (and stores) an individual’s DNA profile to supply personalised nutrition services [13]–[14]. Consumer rejection of nutrigenomics may have concomitant impacts on public health, and result in the commercial failure of a potentially beneficial technology. However, even if putative benefits to individuals and society can be identified, consumer adoption of novel food technologies, including those focused on the improvement of health, should be based on the premise of informed choice [15]. This *a priori* requires the understanding of the psychological and socio-cultural factors which shape consumers perception, attitudes and decision-making related to behaviour. The aim of the research presented here is to develop a predictive model of the psychological factors which predict consumer acceptance or rejection of personalised nutrition.

A focus group study exploring consumer perceptions of, and attitudes towards personalised nutrition was conducted in 8 European countries (Spain; UK; Ireland; Netherlands; Poland; Portugal; Greece; and Germany) [12], the results of which suggested constructs for development of a predictive models of the intention to adopt personalised nutrition. The results indicated that participants framed personalised nutrition in terms of the extent they associated with to be associated with perceived risk, perceived benefit, (which aligns with previous research on personalised nutrition [16]–[18]), the extent to which they were motivated to make dietary changes, and their attitudes toward their own health and expectations regarding regulation of the delivery system.

Perceived personal benefit was identified as a positive attribute of personalised nutrition [16]–[18]. It has been observed that perceived risk and perceived benefit are associated with a range of potentially controversial issues, including those located the health domain and which are inversely correlated. The greater the perceived benefit an individual perceives to be associated with an activity or event, the less risk is proportionally perceived simultaneously [19]–[21]. A similar relationship regarding perceived risks and benefits has been identified in relation to consumer adoption of ICT delivery of goods and services [22]. This would suggest that the greater the perceived benefit, and the less the perceived risk, individuals associate with personalised nutrition, the greater will be their intentions to adopt it. In the exploratory study, negative attitudes were also reported to be associated with internet delivery of personal and identifiable genetic information as well as broad technological issues associated with personal data protection, and, from this, trust in service providers, regulators, legislation put into place to protect privacy and prevent exploitation of consumer data [12]. Social trust in institutions and regulators has been found to be an important determinant of consumer acceptance of technological innovation in the agri-food sector [23]–[26]. Greater consumer trust in those responsible for data protection has also been linked to increased uptake of services which they provide [27]. It is predicted, therefore, that the more individuals trust regulatory systems to optimise consumer protection in relation to nutrigenomics, the greater will be their intentions to adopt personalised nutrition.

Other factors may also be important determinants of consumer uptake of personalised nutrition, and it is important to consider these in the development of a predictive model of individual differences in relation to personalised nutrition in general, and nutrigenomics in particular. The adoption of individualised diets may vary cross-nationally [28]. However, it is quite possible that these do not influence the psychologically (and theoretically) underpinned determinants of whether an individual adopts personalised nutrition – rather they may represent pragmatic barriers to adoption of individualised diets. Comparing populations within (rather than between) EU member countries is useful, as they share a common regulatory regime, “The European Food Law” [29] regarding food safety standards and implementation, reducing the complexity of potentially influential factors [30]–[31], hence the imperative to study factors determining the uptake of personalised nutrition cross-nationally within the EU.

A potentially important determinant of adoption or rejection of personalised nutrition is *Health Locus of Control* [32]. If people believe that they have control over their own health through their own volitional behaviours, they exhibit a high level of Internal Health Locus of Control. External Health Locus of Control relates to the belief that health status is a matter of chance or under the control of powerful others [33]. In practice, research that has looked at the relationship between different Health Locus of Control beliefs and health-related behaviours has reported that only Internal Health Locus of Control beliefs routinely influence health behaviour, in particular in the area of preventative health interventions [34]. It is expected that individuals having a High Internal Health Locus of Control will be more likely to adopt personalised nutrition.

Closely linked to Health Locus of Control is the construct of self-efficacy, which refers to one’s beliefs in capabilities to perform a desired task, such as to cope with test results [35]–[36]. It has been established that self-efficacy can act as a determinant of or mediator between behaviour and intentions [37]–[38], whilst impacting upon goal setting, goal perseverance and behavioural implementation [39]. Self-efficacy can also influence choice of activities, preparation for an activity and effort expended during performance [40]. Empirical evidence and reviews support the relationship between self-efficacy and predictions of health behaviour, including for example, weight control [41]. In-line with theory, therefore, it could be expected that those with high perceived self-efficacy will be more likely to consider adopting personalised nutrition. Those with low perceived self-efficacy may perceive themselves to lack the ability required to complete the activities involved in personalised nutrition.

Another determinant of adoption or rejection of personalised nutrition is the extent to which an individual holds a positive or negative global attitude towards it [17], [42]–[43]. Attitude has been shown to be a reliable predictor of behavioural intention to make certain food choices [44]–[46]. Global attitudes are general evaluations of a broad concept where a positive attitude contributes to intention to adopt specific applications of that concept, i.e. specific personalised nutrition services including a range of specific attributes, and where negative attitudes to the concept of personalised nutrition would make the service less preferable, or, in extreme cases, make consumers categorically reject the service out of hand [47]. Thus, individuals having a more positive attitude towards personalised nutrition should be more likely to adopt it.

To summarise, previous research has identified that the greater the perceptions of benefit, and the lower perceptions of risk, that people perceive to be associated with food innovations targeting health, including personalised nutrition, the more likely they are to subsequently adopt diets based on personalised dietary advice. Research has also indicated that social trust in regulatory intuitions and service providers may influence adoption. Perceived self-efficacy (the extent to which people perceive that adoption of personalised nutrition is achievable) may also be a determinant of adoption. What has not yet been examined is to what extent these different factors (and their potential interrelationships) predict attitudes towards, and intention to adopt, personalised nutrition. In this paper the extent to which perceived risk and benefit associated with adoption of personalised nutrition, perceived self-efficacy, internal locus of control, and social trust influence consumers’ attitudes towards personalised nutrition will be assessed. In turn, the relationship of these factors to self-reported intention to adopt personalised nutrition will also be analysed. The analysis was conducted across 9 EU countries.

## Methods

### Participants and sampling procedure

Newcastle University's Faculty of Science, Agriculture and Engineering's ethics committee identified the research as meeting the criteria for ethical approval. A total of 9381 participants from 9 EU countries (Germany, Greece, Ireland, Poland, Portugal, Spain, the Netherlands, the UK, and Norway) were quota sampled to be nationally representative for each country, on sex, age (18–29, 30–39, 40–54, 55–65 years) and education level (highest level of education completed based on International Standard Classification of Education levels ISCED 0–2, ISCED 3–4, ISCED 5–6). Sample characteristics are summarised by country in Table 1. Participants were drawn from an existing panel of a social research agency. Additional research agencies were subcontracted by the primary agency to supplement panels if needed. A total of 29,450 individuals were contacted, and the overall response rate was 31.9%. Data were collected in February and March 2013, using on-line survey methodology. Information about the research was provided to potential participants in the opening statement of the survey which explained the voluntariness of participation, anonymity of respondents, the purpose of the research, methods employed and funding. Completion of the questionnaire having received the above information was taken as informed consent. An anonymised data set was returned to the researchers following internal data checks.

**Table 1:** Sample Profile.

		Germany	Greece	Ireland	Netherlands	Norway	Poland	Portugal	Spain	UK	TOTAL
		(n = 1020)	(n = 1020)	(n = 1020)	(n = 1020)	(n = 1022)	(n = 1045)	(n = 1148)	(n = 1025)	(n = 1061)	(n = 9381)
**SEX**	**Males** *%*	49.9	49.4	49.8	50.3	52.6	52.1	49.5	51.3	51.0	50.6
**AGE**	**18–29 y** *%*	18.6	24.7	23.5	20.0	20.5	24.4	23.8	19.0	23.0	22.0
	**30–39 y** *%*	16.4	32.1	26.4	18.3	21.6	23.9	25.7	26.6	19.4	23.4
	**40–54 y** *%*	40.5	37.6	32.1	38.2	30.7	28.0	34.8	35.4	36.0	34.8
	**55–65 y** *%*	24.5	5.6	18.0	23.4	27.1	23.6	15.7	18.9	21.6	19.8
**EDUCATION**	**Low** *%*	29.6	31.5	12.2	28.8	38.8	11.2	24.9	32.3	49.0	28.7
	**Middle** *%*	52.9	35.2	37.5	35.6	31.2	61.3	37.9	43.2	15.4	38.9
	**High** *%*	17.5	33.3	50.4	35.6	29.9	27.5	37.2	24.5	35.6	32.4

### Questionnaire development

The results of the exploratory study, [12] together with information from the existing theoretical literature, informed the development of the questionnaire. Validated scales were used to assess *Nutritional Self-Efficacy* and *Health Locus of Control*. Existing validated scales were selected and adapted to measure *Perceived Benefit Associated with Personalised Nutrition*, *Perceived Risk Associated with Personalised Nutrition, Attitudes to Personalised Nutrition*, *Perceived Efficacy of Control and Regulation,* and *Intention to Adopt Personalised Nutrition.*

After the questionnaire had been designed, it was pretested in the UK using face-to-face interviews (n = 16) to determine question comprehension and the length of time needed to complete the questionnaire, and further refined. The revised questionnaire was piloted online in the UK (n = 50), and Portugal (n = 50), using Survey Monkey Software [48]. Minor changes to question order were then introduced to mitigate framing effects, and some question wordings were applied to those items not assessed using validated scales) (see Files S1–S2). The questionnaire was then translated and back-translated into the native languages of each of the countries involved in the study, to ensure consistency in the measurement of constructs.

The following items (also summarised in Table 2) were included in the development of the predictive model, as *a priori* hypothesis had been generated about these. The remaining scales were included on the basis of their explanatory potential, and the results will be reported elsewhere. The items included in the questionnaire, the associated response scales, and source publications are summarised in Table 2.

**Table 2:** Constructs, items and response modes included in the current analysis.

Name of scale	Source	Question asked	Items	Response
**Health locus of control**	Adapted from	Please indicate the extent to which you agree or disagree with the following statements:	-*I can be as healthy as I want to be*	Five point scale: anchored by Completely disagree - Completely agree
***The “internal health locus of control” subscale items are italicised under items. The “Health commitment” subscale items are highlighted in bold under items.***	[32] Gebhardt et al., 2001		*-I am in control of my health*	
			*-I can pretty much stay healthy by taking care of myself*	
			**-Efforts to improve your health are a waste of time (scores reversed before analyses)**	
			**-I am bored by all the attention that is paid to health and disease prevention(scores reversed before analyses)**	
			**-What's the use of concerning yourself about your health you'll only worry yourself to death(scores reversed before analyses)**	
**Risk perception associated with personalised nutrition**	Adapted from	Please indicate the extent to which you agree or disagree with the following statements:	-Personalised nutrition represents a risk to me personally.	Five point scale: anchored by Completely disagree - Completely agree
	[49] Frewer et al., 1994		-Personalised nutrition represents a risk to my family.	
	[50] Frewer et al., 1998		-Personalised nutrition represents a risk to an average member of the society in which I live.	
	[51] Fischer & Frewer, 2009			
	[52] Miles and Scaife, 2003			
	[53] Van Dijk et al., 2011			
**Benefit perception associated with personalised nutrition**	Adapted from	Please indicate the extent to which you agree or disagree with the following statements:	-Personalised nutrition will benefit me personally.	Five point scale: anchored by Completely disagree - Completely agree
	[51] Fischer & Frewer, 2009		-Personalised nutrition will benefit my family.	
	[53] Van Dijk et al., 2011		-Personalised nutrition will benefit an average member of the society in which I live.	
	[54] Verbeke et al., 2001			
**Nutrition self-efficacy**	[55] Schwarzer & Renner, 2000	Please indicate how certain you are that you could overcome the following barriers:	-Even if I need a long time to develop the necessary routines.	Five point scale: anchored by Very uncertain - Very certain
		I can manage to stick to healthy foods:	-Even if I have to try several times until it works.	
			-Even if I have to rethink my entire way of nutrition.	
			- Even if I do not receive a great deal of support from others when making my first attempts.	
			-Even if I have to make a detailed plan.	
**Efficacy of trust and regulation**	Adapted from	I am confident that:	-Current regulations in my country are adequate to protect consumers from the potential risks of personalised nutrition.	Five point scale: anchored by Completely disagree - Completely agree. “I don't know” option (later recoded as “Neither disagree nor agree”)
	[56] De Jonge et al., 2008		-Current regulations in my country are adequate to protect personal data and privacy associated with personalised nutrition.	
	[57] Frewer et al., 1996		-There are adequate procedures in place to ensure that everyone who may benefit from personalised nutrition will have access to services.	
	[25] Poortinga & Pidgeon, 2003			
**Attitude towards personalised nutrition**	Developed from	Personalised nutrition is:	-Worthless to Valuable.	Four individual semantic differential 5-point scales
	[58] Crites et al., 1994		-Unpleasant to Pleasant.	
			-Boring to Interesting.	
			-Bad to Good.	
**Intention to adopt personalised nutrition**	[35] Ajzen, 1991, but adapted for future behaviour,	Please indicate the extent to which you agree or disagree with the following statements:	-I intend to adopt personalised nutrition.	Five point scale: anchored by Completely disagree - Completely agree
	[59] Oliver et al., 1997		-I would consider adopting personalised nutrition.	
	[60] Melnyk et al., 2011		-I am definitely going to adopt personalised nutrition.	

### Data Analysis

**Figure 1:**
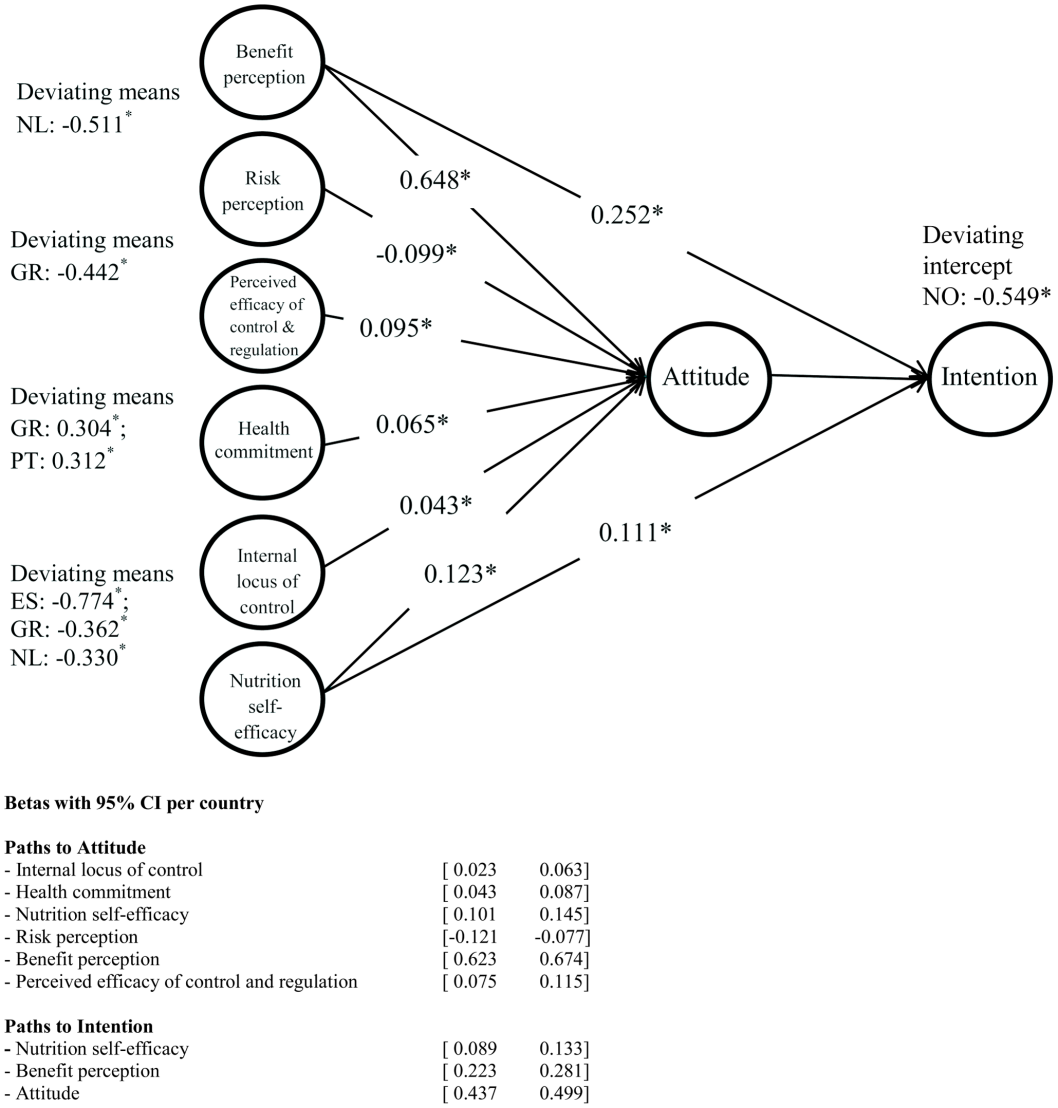
Standardized path coefficients Model.

Quality checks were made by the software used for programming the questionnaire and manually following data extraction. These included, among others, the internal consistency of each construct. All constructs have shown adequate internal consistency. Subsequent analysis focused on the development of multi-group structural equation models conducted in Lavaan [61]. The structural equation model was estimated in three stages. In stage one, the measurement model for each individual construct was assessed. In step two, several models were tested in a stepwise-like procedure, considering direct moderator between constructs, and leading to the model presented in Figure 1. In step three the structural model was estimated. The first and second stage aimed at a cross-cultural validation of the scales, by testing for metric and scalar measurement invariance [62]. Strict measurement invariance was alleviated whenever necessary to ensure that constructs were measured in an equivalent way in all countries. In the final stage, to examine cross-cultural differences, metric and scalar structural invariances were interpreted as indicative of differences between countries. Satorra-Bentler scaled test statistics [63]–[64] were used to accommodate non-normal distributions of the scores on a number of items.

## Results

### Measurement model

Analyses of one factor-models were conducted, for each construct separately. These indicated that the metric invariance across countries could be assumed for all constructs, except *Internal Locus of Control* and *Intention to Adopt Personalised Nutrition* (Table 3). These analyses showed that scalar invariance could be assumed for four out of eight constructs (Nutrition self-efficacy, Risk perception, Benefit perception, and Perceived efficacy of control and regulation), whereas partial scalar invariance seems to hold for the other four constructs (Internal locus of control, Health commitment, Attitude, and Intention to adopt), when adding only few relaxed inequality constraints compared to fully scalar measurement invariance. Based on CFI, TLI and SRMR (Table 3), it was confirmed that all one-factor models demonstrated a good fit when compared to recommended cut-off values (CFI >0.95, TLI>0.95, SRMR<0.08, [65] pp.672) except for *Attitude Towards Personalised Nutrition* where the CFI and TLI were slightly below the cut-off values. The one-factor models for *Nutrition Self-Efficacy, Risk Perception and Benefit Perception* also met the standard for the RMSEA (<0.07) [65], whereas those for *Internal Locus of Control, Health Commitment and Perceived Efficacy of Control and Regulation* are just above the cut-off value, but still below 0.08. For both *Attitude Towards Personalised Nutrition* and *Intention to Adopt Personalised Nutrition* the RMSEA was close to 0.10. Modification indices suggested a two-factor model for *Attitude Towards Personalised Nutrition* consistent with the affective and cognitive component of attitude [66]. However, as for both *Attitude Towards Personalised Nutrition* and *Intention to Adopt Personalised Nutrition* the largest modification indices were comparable to those from the good fitting one-factor models for the other constructs, we decided to give priority to parsimony at this stage and to refrain from further adjustments to their measurement models.

**Table 3:** Fit measures for one-factor models.

One-factor model	Metric invariance	Scalar invariance	Chi-square	Df	CFI	TLI	RMSEA			SRMR
							*Value*	*90% LB*	*90% UB*	
Internal locus of control	Partial^a^	Partial^a^	189.79	26	0.968	0.967	0.078	0.069	0.087	0.036
Health commitment	Yes	Partial^b^	186.41	28	0.966	0.967	0.074	0.064	0.083	0.039
Risk perception associated with personalised nutrition	Yes	Yes	72.72	32	0.998	0.998	0.035	0.026	0.044	0.015
Benefit perception associated with personalised nutrition	Yes	Yes	151.61	32	0.989	0.990	0.060	0.051	0.069	0.026
Nutrition self-efficacy	Yes^c^	Yes^c^	494.12	108	0.978	0.982	0.059	0.054	0.063	0.038
Perceived efficacy of control and regulation	Yes	Yes	204.94	32	0.976	0.980	0.072	0.064	0.081	0.034
Attitude towards personalised nutrition	Yes	Partial^d^	723.36	65	0.929	0.941	0.099	0.093	0.104	0.065
Intention to adopt personalised nutrition	Partial^e^	Partial^e^	297.99	25	0.977	0.976	0.102	0.092	0.113	0.044

a Equality of item loadings (and intercepts) relaxed for third item in Spain and Greece. Equality of item intercept relaxed for first item in Poland and for third item in Portugal.

b Equality of item intercepts relaxed for second item in Norway and for third item in Spain, Greece, and The Netherlands.

c Model includes error covariance between first and second item, equal across countries.

d Equality of item intercept relaxed for third item in The Netherlands.

e Equality of item loading (and intercept) relaxed for second item in Spain. Equality of item intercept relaxed for first item in Greece, for second item in Norway, Germany, and the Netherlands, and for third item in Germany.

The (partial scalar) multi-factor model included all eight constructs simultaneously (with the relaxed equality constraints based on the one-factor models) and demonstrated very good fit on the CFI, TLI, RMSEA, and SRMR (Table 4), when compared to the suggested cut-off values (CFI>0.92, TLI>0.92, RMSEA<0.07, SRMR<0.08) [65].

**Table 4:** Fit measures for multi-factor model and structural equation models.

	Chi-square	Df	CFI	TLI	RMSEA			SRMR
					*Value*	*90% LB*	*90% UB*	
**Multi-factor model**								
Partial scalar measurement invariance^a^	7731.5	2949	0.964	0.961	0.039	0.039	0.040	0.041
**Structural equation models**								
*i*. Configural structuralinvariance^a^	7921.5	2985	0.963	0.960	0.040	0.039	0.041	0.043
*Metric structural invariance*								
*ii*. equal path coefficients^a^	8146.2	3057	0.962	0.960	0.040	0.039	0.041	0.050
*iii*. also partially equal (co-) variances among exogenous LV’s^ab^	9050.6	3224	0.956	0.957	0.042	0.041	0.043	0.083
*Scalar structural invariance*								
*iv*. equal regression intercepts^abc^	9252.3	3239	0.955	0.956	0.042	0.041	0.043	0.084
*v*.+equal means exogenous LV’s^abcd^	10022.1	3280	0.949	0.951	0.044	0.044	0.045	0.090
*vi*.+equal R^2^ Attitude^abcd^	10046.3	3288	0.949	0.951	0.044	0.044	0.045	0.091

a Relaxations on item loadings and intercepts adopted from one-factor measurement models (see footnotes Table 3).

b Equality restriction relaxed for covariance between Risk Perception and Benefit Perception in The Netherlands (see footnotes Table 3).

c Equality restriction relaxed for regression intercept for Intention in Norway (see Figure 1).

d Equality restrictions relaxed for means of Internal Locus of Control in Spain, Greece, and The Netherlands, for Involvement with Health Improvement in Portugal and Greece, for Benefit Perception in The Netherlands, and Perceived Efficacy Control/Regulations in Greece (see Figure 1).

### Structural model

After the measurement model had been consolidated, the hypothesized structural model was tested and regression parameters estimated (Figure 1). First, configural structural invariance across countries was tested (Model *i*), after which cross-country equality constraints were consecutively added on: *ii*) the path coefficients, *iii*) variances and covariances among the six exogenous latent variables, *iv*) intercepts for the regression equations for *Attitude towards Personalised Nutrition* and *Intention to Adopt Personalised Nutrition, v*) means of the six exogenous latent variables, and *vi*) the R^2^ (or equivalently, the disturbance terms) for the regression equation for *Intention to Adopt Personalised Nutrition*. Table 4 provides the fit measures for the six models. Only a few constraints had to be relaxed in Model *iii*, *iv* and *v*. The final model (Model *vi*) shows that few modifications compared to full scalar structural invariance were necessary to obtain a good fitting model. Of the recommended fit measures only the SRMR was higher than recommended, indicating that there was some lack of fit which was compensated by the parsimony of the model.

Model-based internal consistency reliabilities (α) [67]–[68] except for the *Internal Locus of Control* in Spain were higher than the recommended cut-off value of 0.7 (Table 5), and most reliabilities were above 0.8. In the case of *Internal Locus of Control* in Spain, one item (“I am in control of my health”) had a very low correlation with the other two items in the scale and, therefore, the equality constraint on its loading was relaxed at the step where the one-factor model was tested (Table 3).

**Table 5:** Model-based internal-consistency reliabilities Model *vi.*

Construct	Country								
	Norway	Germany	Spain	Greece	Poland	UK	Ireland	Netherlands	Portugal
Internal locus of control	0.810	0.844	0.687	0.780	0.726	0.846	0.818	0.847	0.779
Health commitment	0.757	0.740	0.790	0.774	0.789	0.782	0.781	0.771	0.839
Risk perception associated with personalised nutrition	0.966	0.956	0.956	0.966	0.964	0.968	0.962	0.974	0.973
Benefit perception associated with personalised nutrition	0.924	0.931	0.949	0.932	0.945	0.942	0.946	0.965	0.968
Nutrition self-efficacy	0.874	0.879	0.881	0.872	0.887	0.897	0.876	0.900	0.899
Perceived efficacy of control and regulation	0.871	0.860	0.858	0.865	0.882	0.893	0.841	0.895	0.867
Attitude towards personalised nutrition	0.855	0.880	0.833	0.872	0.885	0.865	0.855	0.897	0.885
Intention to adopt personalised nutrition	0.947	0.943	0.899	0.887	0.919	0.912	0.905	0.953	0.919

The correlations between the exogenous latent variables were as expected. For instance, *Risk* and *Benefit Perception* were negatively correlated (r = −0.172) (except for the Netherlands, r = 0.296) (Table 6). The larger correlations were found between *Nutrition Self-Efficacy* and *Internal Locus of Control* (r = 0.368), *Nutrition Self-Efficacy* and *Benefit Perception* (r = 0.307), and between scores on the *Health Commitment* sub-scale and *Risk Perception* (r = −0.293). A relevant proportion of variance (R^2^) in *Attitudes towards Personalised Nutrition* and *Intention to Adopt Personalised Nutrition* was explained by the model in all countries (Table 7). Figure 1 provides the standardized path coefficients for the Netherlands, as well as the means of exogenous latent variables and regression intercept deviating from the overall means and intercepts, which were set equal to zero for identification purposes. Standardized path coefficients in the structural equation for *Attitude towards Personalised Nutrition* are exactly the same in other countries, because the proportion of variances accounted for in attitudes were constrained to be equal across countries (which did not deteriorate the model, see the similarity in fit between structural equation models *v* and *vi* in Table 4). Standardized path coefficients in the structural equation for *Intention to Adopt Personalised Nutrition* differed between countries proportional to differences in R^2^, with the R^2^ in the Netherlands being closest to the mean R^2^.

**Table 6:** Correlations among exogenous latent variables in Model vi.

Construct	Construct				
	Internalhealthlocus ofcontrol	Healthcommitment	Risk perceptionassociated withpersonalisednutrition	Benefit perceptionassociated withpersonalisednutrition	Nutritionself-efficacy
Internal locus of control health					
Health commitment	0.107*				
Risk perception associated withpersonalised nutrition	−0.021#	−0.293*			
Benefit perception associatedwith personalised nutrition	0.145*	0.197*	−0.172* NL: 0.296*		
Nutrition self-efficacy	0.368*	0.213*	−0.002#	0.307*	
Perceived efficacy control/regulationsassociated with personalised nutrition	0.151*	−0.062*	0.081*	0.151*	0.135*

# p>0.05; * p<0.001.

**Table 7:** Proportion of variance accounted for (R^2^) structural equations in Model vi.

Construct	Country								
	Norway	Germany	Spain	Greece	Poland	UK	Ireland	Netherlands	Portugal
Attitude towards personalised nutrition^a^	0.555	0.555	0.555	0.555	0.555	0.555	0.555	0.555	0.555
Intention to adopt personalised nutrition	0.371	0.463	0.479	0.676	0.528	0.499	0.544	0.514	0.545

a R^2^ equal across countries because of equality constraints.

While all hypothesized relations were significant, it was obvious that both *Attitude towards Personalised Nutrition* and *Intention to Adopt Personalised Nutrition* depend most on people’s *Benefit Perception.* The second strongest effect comes from the *Nutrition self-efficacy scale*, which has a (less strong) positive relationship with both *Attitude towards Personalised Nutrition* and *Intention to Adopt Personalised Nutrition.* The results show only few differences in means between countries. The mean *Benefit perception* was somewhat lower in The Netherlands compared to other countries. The mean *Perceived Efficacy of Control and Regulation* was somewhat lower in Greece than in other countries. The mean *Health Commitment* was somewhat lower in Greece and Portugal. In Spain, the mean *Internal Locus of Control* was the lowest and in Greece and The Netherlands, somewhat lower than in other countries. Compared to other countries, the mean *Intention to Adopt Personalised Nutrition* was half a scale point lower in Norway than one would expect on the basis of the mean scores on all other constructs.

## Discussion

*Benefit Perception* had a direct relationship with both *Intention to Adopt Personalised Nutrition*, and overall *Attitude towards Personalised Nutrition*. As predicted from the literature, an inverse relationship was observed between *Perceived Benefit* and *Risk Associated with Personalised Nutrition*. *Perceived Risk* had a weaker influence on *Attitude towards Personalised Nutrition*, and no direct relationship with *Intention to Adopt Personalised Nutrition.* The positive relationship between *Perceived Benefit,* and both *Attitudes towards,* and *Intention to adopt, Personalised Nutrition,* was intuitive. The risk perception literature would imply that perceived risk would be more likely to predict consumer rejection and that perceived benefit would predict consumer acceptance [21], [69] although, see [70]. This observation is in line with the prior qualitative results of Stewart-Knox *et al.* (2013), which indicated that Perceived Risk was not *intrinsically* associated with personalised nutrition, but rather reflected broader concerns associated with the delivery system for personalised nutrition services (for example, with regard to the extent to which the internet was perceived to represent a secure means of transmitting and storing an individual’s genetic, or even phenotypic data). Thus consumers appeared to have more positive attitudes towards, and expressed greater intention to adopt personalised nutrition under circumstances where they perceived that personalised nutrition would deliver benefits, and that these benefits would be achievable.

*Nutrition Self-Efficacy* also exhibited a positive relationship with both *Attitude* and *Intention to adopt Personalised Nutrition*. Consistent with Social Learning Theory [40], the higher an individual was in self-efficacy, the greater the expectation that they would successfully engage in dietary behaviour change. The findings corroborate previous research which has suggested that greater self-efficacy is associated with perceived ability to make healthy food choices [61]–[73], intention to make healthy food choices [74]–[75] and achievement of healthier dietary habits [71], [72], [76], [77].

Attitude has been shown to be a reliable predictor of behavioural intention regarding food choices [78]. Attitude incorporates global and abstract evaluations of risks and benefits, and can be differentiated from intentions which appear to be based primarily on concrete and tangible benefits ([79], Fischer, *et al*., unpublished data). The research reported here suggests that holding a positive attitude towards personalised nutrition, high perceived self-efficacy and perception of personal benefit associated with personalised nutrition appear to contribute directly to intention to take up personalised nutrition.

A positive relationship was also found to exist between *Internal Health Locus of Control* and *Attitude towards Personalised Nutrition,* which is in line with that for *Nutrition Self-Efficacy*. The few studies that have considered how health locus of control affects dietary related behaviours, have suggested that higher internal health locus of control is associated with better knowledge of nutrition [80] and greater perceived importance of nutrition over taste or convenience when selecting foods [81]. The results are also consistent with the notion of interplay between perceived control and self-efficacy [36].

Thus the more people perceived that their own actions and behaviours could potentially have a positive impact on their own health status, and the more they perceived that this could be achieved through dietary choices, the more likely they would be to hold a positive attitude towards personalised nutrition. *Perceived Efficacy of Control and Regulation* was also positively related to attitude, and again one might expect this to be the case given the “risk based” concerns identified in [12] being linked primarily to data storage and confidentiality concerns, rather than factors *intrinsic* to the nutrigenomics technology. These concerns may be mitigated by application and identification of efficacious, transparent and enforced regulatory and governance practices. Finally, and in a similar vein, the extent to which participants expressed high levels of “health commitment” (using the health commitment items from Gebhardt et al., (2001) the *External Health Locus of Control Scale*), the more positive their attitude towards personalised nutrition. In summary, those individuals who perceived most benefits to be associated with personalised nutrition, perceived that they could achieve these health goals, and those who had greatest trust in those regulatory and control systems designed to promote consumer protection were the most likely to adopt personalised nutrition.

Some recommendations for developing communication about personalised nutrition can be identified. First, the results suggest that people will be primarily interested in receiving information about potential (and personal) benefits of adopting personal nutrition. Although benefits (and consumer recognition of these) are very important as a determinant of consumer acceptance of personalised nutrition, the form that these take may vary considerably between different consumers, and may need to reflect the individual goals which consumers are interested. Second, information about ease of adoption of personalised nutrition may convince potential adopters not only of the benefits, but the attainability of these, thus increasing perceptions of self-efficacy. Some individuals may be reinforced in their commitment by internet based coaching, while others may prefer a directly personalised approach using meeting with health professionals [12]. Third, transparent regulations regarding protection of data, in particular, but not exclusively genomic data, are required. There needs to be evidence of enforcement of these regulations across both the private and public sectors and information about these needs to be communicated to the public. In order to develop trust, it is also necessary to engage with the public regarding the design of legislative infrastructure and subsequent implementation of regulations, a debate which is likely to extend beyond personalised nutrition to other areas of personalised medicine, including regulations designed to promote data protection.

The psychological determinants of consumer acceptance of personalised nutrition were relatively stable across the different EU countries involved in this research. However, although perceived benefit and self-efficacy may be important determinants of uptake of personalised nutrition across all 9 European countries involved in the study, there may be considerable local variation in what constitutes the facilitators of, or barriers to, adoption of personalised nutrition. This may relate to local infrastructure (e.g. the perceived efficiency and effectiveness of local postal services, in relation to delivering blood samples or receiving confidential medical information, and funding for local health service provision) [12]. It may also reflect local socio-cultural variations in food choices (e.g. the extent to which people eat meals comprising the same ingredients in extended families or social groups), making it difficult to take account of individual differences in food choices prescribed by personalised dietary advice. The lack of invariance in the model, however, limits the degree to which it is possible to compare differences across countries. The observation that different facilitators and barriers would lead to equal levels of perceived self-efficacy across countries, therefore, needs further explanation.

Some limitations of the research reported here can be identified. Despite the large sample, the compliance rate achieved (31.9%) could somewhat constrain the generalization of results. Nevertheless, the sampling procedure, namely quota sampling to achieve national representativeness for each country, reduces the potential impact associated with this limitation. Not all multi-item scales used for measuring the constructs in our model exhibited complete scalar measurement invariance, which may cast some doubt on measurement equivalence [82]. The robustness of the model across different countries suggests that our approach, assuming only partial measurement invariance by alleviating restrictions, was appropriate enough to overcome this problem. Future research investigating construct equivalence and developing multi-item scales with better measurement equivalence would be necessary to tackle the problem at the source.

Another limitation of this study is that no measure of actual behaviour has been included. The concept of personalised nutrition is relatively new. It is, as a consequence, not yet available at the public health level and so it was assumed that few respondents would have experience of personalised nutrition. The intention to adopt personalised nutrition therefore, refers to a hypothetical concept rather than actual behaviour for many individuals. Expectancy value theories [36] [40], [83]–[85], suggest that intention to adopt as in these results have direct implications for behaviour change. The Theory of Planned Behaviour [35] postulates that behaviour is an outcome of attitude, social norms, and perceived behavioral control related to the object (e.g. personalised nutrition) and intention to execute the behaviour (e.g. engage in personalised nutrition) [35]. Some elements of the Theory of Planned Behaviour, specifically those related to attitude and intention and perceived self -efficacy have been included in this analysis. Among the main findings of the present study was that the perceived efficacy of regulatory control to protect data from misuse was associated with attitudes toward and intention to take up personalised nutrition. Given evidence from previous studies suggesting that intention is related to health behaviour change [35], [38], findings from the present study could imply that benefits associated with personalised nutrition, risk from misuse of health data and confidence in regulation of on-line privacy and data handling etc. are likely to predict uptake of personalised nutrition.

Protection Motivation Theory [83]–[85] also considers the perceived costs and benefits of taking remedial action to reduce risk but goes further to suggest that action is a function of the perceived size and severity of an event, the likelihood that an event will occur and ability to respond effectively to reduce the risk. Self-efficacy which is a construct integral to Social Cognitive Theory [36], [40] and which has been measured as part of the present study, has been shown to correlate with these elements of risk perception comprising Protection Motivation Theory [83]. Perceived size, severity and likelihood of an event and ability to respond to the event together with self-efficacy have been shown to moderate attitude and intention to change behaviour [85] and this could have implications for the application of the results to practice and future research. Uptake of personalised nutrition may depend on societally approved and transparent regulation of on-line data use and the development of more effective data protection technologies, as well as communication to the public about high levels of data security applied, although further research is required to determine if information about data security and potential risk mitigation measures increases end-user uptake of personalised nutrition services. On the basis of this analysis, it can be hypothesised that individuals who have actually adopted personalised nutrition will score higher on perceived self-efficacy, perceived benefit and lower on perceived risk and involvement, and exhibit a high internal locus of control. This hypothesis will be explored in future research, where data similar to those collected in the current survey will be obtained from participants taking part in a personalised nutrition trial, also being conducted within the Food4Me project in seven of the same countries involved in the survey.

## Conclusions

To the authors’ knowledge, this is the first study to have modelled factors determining intention to take up personalised nutrition in representative samples of European consumers. An important strength of the study was that the elements of the model have been informed by qualitative research in similar population. These data imply that attitudes towards, and adoption of, personalised nutrition are primarily driven by perceptions of benefit and whether adoption of personalised nutrition is achievable. Trust in regulatory systems (in particular related to data protection) and the extent to which individuals are committed to improving, and perceive that their own actions may influence their own health status and attitudes towards personalised nutrition. This implies that promotion of personalised nutrition to the general public would need to emphasise the (personal) benefits of personalised nutrition. Discussion of risk should focus on end-user concerns, in particular related to data –protection and service delivery. Communication should also address Perceived Efficacy through providing information about how personalised nutrition can be adopted by consumers. Providing information about potential health benefits associated with personalised nutrition may also influence adoption by individuals with low levels of Health Locus of Control.

## Supporting Information

File S1 **Consumer attitudes towards personalised nutrition questionnaire.** The items included in the current analyses are presented, together with additional items.
(DOCX)

File S2 **Data File (SPSS format, data relevant to current analysis).**
(HTM)
