# Supplementary material for: Psychological Determinants of Consumer Acceptance of Personalised Nutrition in 9 European Countries
Source: PLoS One. 2014 Oct 21;9(10):e110614. doi: 10.1371/journal.pone.0110614 (PMC4204923; doi:10.1371/journal.pone.0110614)
Supplement: File S1 — Consumer attitudes towards personalised nutrition questionnaire. The items included in the current analyses are presented, together with additional items. (DOCX) [file pone.0110614.s001.docx]

Dear participant,

Thank you for taking the time to fill in this questionnaire with regard to your views on personalised nutrition.

Your participation is totally voluntary. Please remember we are seeking your opinions and there are no right or wrong answers. All your answers will be kept completely anonymous. The questions take about 20 minutes to answer.

This survey is part of the EU funded project “Food4Me” which is examining the issues and challenges associated with personalised nutrition. One of the objectives of the Food4Me project is to understand consumers’ opinions about personalised nutrition.

Before you start answering the questions we would like to draw your attention to the definition of personalised nutrition which is: **“healthy eating advice that is tailored to suit an individual based on their own personal health status, diet, physical activity and/or genetics”**

Thank you for your participation!

|  | | | | | | | |
| --- | --- | --- | --- | --- | --- | --- | --- |
| **Q1. Please indicate the extent to which you agree or disagree with the following statements:** | | | | | | | |
|  |  | *Completely disagree* | *Disagree* | *Neither disagree/nor agree* | *Agree* | *Completely agree* |  |
| I can be as healthy as I want to be |  | O | O | O | O | O |  |
| I am in control of my health |  | O | O | O | O | O |  |
| I can pretty much stay healthy by taking care of myself |  | O | O | O | O | O |  |
| Efforts to improve your health are a waste of time |  | O | O | O | O | O |  |
| I am bored by all the attention that is paid to health and disease prevention |  | O | O | O | O | O |  |
| What's the use of concerning yourself about your health you'll only worry yourself to death |  | O | O | O | O | O |  |

|  | | | | | | | |
| --- | --- | --- | --- | --- | --- | --- | --- |
| **Q2. Please indicate the extent to which you agree or disagree with the following statements:** | | | | | | | |
|  |  | *Completely disagree* | *Disagree* | *Neither disagree/nor agree* | *Agree* | *Completely agree* |  |
| Eating healthily is something I do frequently |  | O | O | O | O | O |  |
| I eat healthily without having to consciously think about it |  | O | O | O | O | O |  |
| I feel weird if I don’t eat healthily |  | O | O | O | O | O |  |
| Eating healthily is something I do without having to think about it doing |  | O | O | O | O | O |  |

|  | | | | | | | | |
| --- | --- | --- | --- | --- | --- | --- | --- | --- |
| **Q3. Please indicate how certain you are that you could overcome the following barriers:** | | | | | | | | |
|  |  | *Very uncertain* | *Uncertain* | *Neither certain/nor uncertain* | *Certain* | *Very certain* | |  |
| I can manage to stick to healthy foods: even if I need a long time to develop the necessary routines |  | O | O | O | O | O |  | |
| I can manage to stick to healthy foods: even if I have to try several times until it works |  | O | O | O | O | O |  | |
| I can manage to stick to healthy foods: even if I have to rethink my entire way of nutrition |  | O | O | O | O | O |  | |
| I can manage to stick to healthy foods: even if I do not receive a great deal of support from others when making my first attempts |  | O | O | O | O | O |  | |
| I can manage to stick to healthy foods: even if I have to make a detailed plan |  | O | O | O | O | O |  | |

| **Q4. Please give your position on the following statements.**  **It is important to me that the food I eat on a typical day:** | | | | | |
| --- | --- | --- | --- | --- | --- |
|  | *Not at all important* | *A little important* | *Moderately Important* | *Very Important* | *Extremely important* |
| Contains a lot of  vitamins and minerals | O | O | O | O | O |
| Keeps me healthy | O | O | O | O | O |
| Is nutritious | O | O | O | O | O |
| Is high in protein | O | O | O | O | O |
| Is good for my  skin/teeth/hair/nails etc. | O | O | O | O | O |
| Is high in fibre and roughage | O | O | O | O | O |
| Helps me cope with stress | O | O | O | O | O |
| Helps me to cope with life | O | O | O | O | O |
| Helps me relax | O | O | O | O | O |
| Keeps me awake/alert | O | O | O | O | O |
| Cheers me up | O | O | O | O | O |
| Makes me feel good | O | O | O | O | O |
| Is easy to prepare | O | O | O | O | O |
| Can be cooked very simply | O | O | O | O | O |
| Takes no time to prepare | O | O | O | O | O |
| Can be bought in shops close to  where I live or work | O | O | O | O | O |
| Is easily available in shops and supermarkets | O | O | O | O | O |
| Smells nice | O | O | O | O | O |
|  | *Not at all important* | *A little important* | *Moderately important* | *Very Important* | *Extremely important* |
| Looks nice | O | O | O | O | O |
| Has a pleasant texture | O | O | O | O | O |
| Tastes good | O | O | O | O | O |
| Contains no additives | O | O | O | O | O |
| Contains natural ingredients | O | O | O | O | O |
| Contains no artificial ingredients | O | O | O | O | O |
| Is not expensive | O | O | O | O | O |
| Is cheap | O | O | O | O | O |
| Is good value for money | O | O | O | O | O |
| Is low in calories | O | O | O | O | O |
| Helps me control my weight | O | O | O | O | O |
| Is low in fat | O | O | O | O | O |
| Is what I normally eat | O | O | O | O | O |
| Is well-known | O | O | O | O | O |
| Is like the food I ate when I was a child | O | O | O | O | O |
| Comes from countries I approve of politically | O | O | O | O | O |
| Has the country of origin clearly marked | O | O | O | O | O |
| Is packaged in an environmentally friendly way | O | O | O | O | O |

| **Q5. Please indicate the extent to which you agree or disagree with the following statements:** | | | | | | | |
| --- | --- | --- | --- | --- | --- | --- | --- |
|  |  | *Completely disagree* | *Disagree* | *Neither disagree/nor agree* | *Agree* | *Completely agree* |  |
| Personalised nutrition represents a risk to me personally |  | O | O | O | O | O |  |
| Personalised nutrition represents a risk to my family |  | O | O | O | O | O |  |
| Personalised nutrition represents a risk to an average member of the society in which I live |  | O | O | O | O | O |  |

|  | | | | | | | |
| --- | --- | --- | --- | --- | --- | --- | --- |
| **Q6. Please indicate the extent to which you agree or disagree with the following statements:** | | | | | | | |
|  |  | *Completely disagree* | *Disagree* | *Neither disagree/nor agree* | *Agree* | *Completely agree* |  |
| Personalised nutrition will benefit me personally |  | O | O | O | O | O |  |
| Personalised nutrition will benefit my family |  | O | O | O | O | O |  |
| Personalised nutrition will benefit an average member of the society in which I live |  | O | O | O | O | O |  |

| **Q7. Personalised nutrition is:** | | | | | | | | |
| --- | --- | --- | --- | --- | --- | --- | --- | --- |
|  |  | *Very worthless* | *Worthless* | *Neither worthless/nor valuable* | *Valuable* | *Very valuable* |  |  |
| **Worthless** |  | O | O | O | O | O |  | **Valuable** |
|  |  |  |  |  |  |  |  |  |
|  |  | *Very unpleasant* | *Unpleasant* | *Neither unpleasant/nor pleasant* | *Pleasant* | *Very pleasant* |  |  |
| **Unpleasant** |  | O | O | O | O | O |  | **Pleasant** |
|  |  |  |  |  |  |  |  |  |
|  |  |  |  |  |  |  |  |  |
|  |  | *Very boring* | *Boring* | *Neither boring/nor interesting* | *Interesting* | *Very interesting* |  |  |
| **Boring** |  | O | O | O | O | O |  | **Interesting** |
|  |  |  |  |  |  |  |  |  |
|  |  | *Very bad* | *Bad* | *Neither bad/nor good* | *Good* | *Very good* |  |  |
| **Bad** |  | O | O | O | O | O |  | **Good** |

| **Q8. Please indicate the extent to which you agree or disagree with the following statements:** | | | | | | | |
| --- | --- | --- | --- | --- | --- | --- | --- |
|  |  | *Completely disagree* | *Disagree* | *Neither disagree/nor agree* | *Agree* | *Completely agree* |  |
| I intend to adopt personalised nutrition |  | O | O | O | O | O |  |
| I would consider adopting personalised nutrition |  | O | O | O | O | O |  |
| I am definitely going to adopt personalised nutrition |  | O | O | O | O | O |  |

| **Q9. How appealing is personalised nutrition based upon:** | | | | | | | |
| --- | --- | --- | --- | --- | --- | --- | --- |
|  |  | *Not at all appealing* | *Slightly appealing* | *Moderately appealing* | *Very appealing* | *Extremely appealing* |  |
| Information regarding the food you eat and the exercise you take |  | O | O | O | O | O |  |
| Your blood chemistry in addition to information regarding the food you eat and the exercise you take |  | O | O | O | O | O |  |
| An analysis of your DNA in addition to information regarding the food you eat and the exercise you take |  | O | O | O | O | O |  |

| **Considering that a diet program provided by a qualified dietician costs about 100 € for the first 6 months.**  **Q10. Would you be willing to pay 100 € for personalised nutrition based upon information you have provided about the food you eat and the exercise you take?**  O Yes (answer question A and go to Q21)  O No (answer questions B and C and go to Q21)  **If YES:**  **A.** How much would you be willing to pay at most for this service as a maximum? Please answer on the scale below by drawing a cross*.   \| 100 euro \|  \| 500  euro \| \| --- \| --- \| --- \|   ----* For the digital version of the questionnaire plaese make sure that the sliding cursor is situated at the left end (100 euro) of the bar.----  **If NO:**  **B.** How much would you be willing to pay for this service as a maximum? Please answer on the scale below by drawing a cross*.   \| 0 euro \|  \| 99  euro \| \| --- \| --- \| --- \|   ----* For the digital version of the questionnaire plaese make sure that the sliding cursor is situated at the right end (99 euro) of the bar.-----  **C.** What reason is most important for you in wanting to pay less for personalised nutrition based upon information you have provided about the food you eat and the exercise you take compared to conventional dietary advice, tick any which apply:  O I cannot afford to pay  O I can get this for free  O My GP/health provider should provide this for free  O I am not interested in personalised nutrition  O I do not think it will be useful to me  O I don’t think it is possible to provide personalised nutrition on the basis of information  regarding the food I eat and the exercise I take  O Other, please specify……………………………………………………………………………………….. …………..…………………………………………………………………………………………………………  **Q11. Would you be willing to pay 100 € for personalised nutrition based upon an analysis of your blood chemistry in addition to information you have provided about the food you eat and the exercise you take?**  O Yes (answer question D and go to Q22)  O No (answer questions E and F and go to Q22)  **If YES:**  **D.** How much would you be willing to pay at most for this service as a maximum? Please answer on the scale below by drawing a cross*.   \| 100 euro \|  \| 500  euro \| \| --- \| --- \| --- \|   ----* For the digital version of the questionnaire plaese make sure that the sliding cursor is situated at the left end (100 euro) of the bar.-----  **If NO:**  **E.** How much would you be willing to pay for this service as a maximum? Please answer on the scale below by drawing a cross*.   \| 0 euro \|  \| 99  euro \| \| --- \| --- \| --- \|   ----* For the digital version of the questionnaire plaese make sure that the sliding cursor is situated at the right end (99 euro) of the bar.-----  **F.** What reason is most important for you in wanting to pay less for personalised nutrition based upon an analysis of your blood chemistry in addition to information you have provided about the food you eat and the exercise you take, tick any which apply:  O I cannot afford to pay  O I can get this for free  O My GP/health provider should provide this for free  O I am not interested in personalised nutrition  O I do not think it will be useful to me  O I don’t think it is possible to provide personalised nutrition on the basis an analysis of my blood  chemistry in addition to information I have provided about the food I eat and the exercise I take  O Other, please specify…………………………………………………………………………………………. ………..……………………………………………………………………………………………………………..  **Q12. Would you be willing to pay 100 € for personalised nutrition based upon an analysis of a sample of your DNA in addition to information you have provided about the food you eat and the exercise you take?**  O Yes (answer question G and go to Q23)  O No (answer questions H and I and go to Q23)  **If YES:**  **G.** How much would you be willing to pay at most for this service as a maximum? Please answer on the scale below by drawing a cross*.   \| 100 euro \|  \| 500  euro \| \| --- \| --- \| --- \|   ----* For the digital version of the questionnaire plaese make sure that the sliding cursor is situated at the left end (100 euro) of the bar.-----  **If NO:**  **H.** How much would you be willing to pay for this service as a maximum? Please answer on the scale below by drawing a cross*.   \| 0 euro \|  \| 99  euro \| \| --- \| --- \| --- \|   ----* For the digital version of the questionnaire plaese make sure that the sliding cursor is situated at the right end (99 euro) of the bar.----  **I.** What reason is most important for you in wanting to pay less for personalised nutrition based upon an analysis of a sample of your DNA in addition to lifestyle information, tick any which apply:  O I cannot afford to pay  O I can get this for free  O My GP/health provider should provide this for free  O I am not interested in personalised nutrition  O I do not think it will be useful to me  O I don’t think it is possible to provide personalised nutrition on the basis an analysis of a  sample of my DNA in addition to information I have provided about the food I eat and the exercise I  take  O Other, please specify…………………………………………………………………………………………… ………………………………………………………………………………………………………….…………….. |
| --- | --- | --- | --- | --- | --- | --- | --- | --- | --- | --- | --- | --- | --- | --- | --- | --- | --- | --- |

|  | | | | | | | |
| --- | --- | --- | --- | --- | --- | --- | --- |
| **Q13. I am confident that:** | | | | | | | |
|  |  | *Completely disagree* | *Disagree* | *Neither disagree/ nor agree* | *Agree* | *Completely agree* | I don’t know |
| Current regulations in my country are adequate to protect consumers from the potential risks of personalised nutrition |  | O | O | O | O | O | O |
| Current regulations in my country are adequate to protect personal data and privacy associated with personalised nutrition |  | O | O | O | O | O | O |
| There are adequate procedures in place to ensure that everyone who may benefit from personalised nutrition will have access to services |  | O | O | O | O | O | O |

| **Q14. Please indicate the extent to which you trust each of the following organisations to protect consumers in relation to personalised nutrition services:** | | | | | | | |
| --- | --- | --- | --- | --- | --- | --- | --- |
|  |  | *Distrust extremely* | *Distrust* | *Neither trust/nor distrust* | *Trust* | *Trust extremely* |  |
| Your national government ministry or department of health |  | O | O | O | O | O |  |
| The European Commission |  | O | O | O | O | O |  |
| Your health provider **(adjust to national requirements)** |  | O | O | O | O | O |  |
| Food manufacturers |  | O | O | O | O | O |  |
| Food retailers |  | O | O | O | O | O |  |
| Consumer organisations |  | O | O | O | O | O |  |
| Universities |  | O | O | O | O | O |  |
| Health insurance companies |  | O | O | O | O | O |  |
| Other, please specify ………………………………………………………………………………………………  ………………………………………………………………………………………………………………………… | | | | | | | |

|  | | | | | | | |
| --- | --- | --- | --- | --- | --- | --- | --- |
| **Q15. Please indicate the extent to which you trust each of the following information sources to provide accurate information about personalised nutrition**: | | | | | | | |
|  |  | *Distrust extremely* | *Distrust* | *Neither trust/n or distrust* | *Trust* | *Trust extremely* |  |
| Your family doctor |  | O | O | O | O | O |  |
| Your national government ministry or department of health |  | O | O | O | O | O |  |
| The European Commission |  | O | O | O | O | O |  |
| Your health provider **(adjust to national requirements)** |  | O | O | O | O | O |  |
| Food retailers |  | O | O | O | O | O |  |
| Food manufacturers |  | O | O | O | O | O |  |
| Online personalised nutrition companies |  | O | O | O | O | O |  |
| Universities |  | O | O | O | O | O |  |
| Consumer organizations |  | O | O | O | O | O |  |
| Dieticians/nutritionists |  | O | O | O | O | O |  |
| Personal trainers |  | O | O | O | O | O |  |
| Friends and family |  | O | O | O | O | O |  |
| News media |  | O | O | O | O | O |  |
| Social media |  | O | O | O | O | O |  |
| Other, please specify ………………………………………………………………………………………………  ………………………………………………………………………………………………………………………… | | | | | | | |
|  |  |  |  |  |  |  |  |

|  | | | | | | | |
| --- | --- | --- | --- | --- | --- | --- | --- |
| **Q16. Please indicate the extent to which the following potential outcomes would increase the likelihood of you adopting personalised nutrition:** | | | | | | | |
|  |  | *Not increase it at all* | *Increase it slightly* | *Increase it moderately* | *Increase it strongly* | *Increase it extremely* |  |
| Knowing what foods are best for me |  | O | O | O | O | O |  |
| Losing weight |  | O | O | O | O | O |  |
| Gaining weight |  | O | O | O | O | O |  |
| Fitness |  | O | O | O | O | O |  |
| Improving my family's health |  | O | O | O | O | O |  |
| Improving my health |  | O | O | O | O | O |  |
| Improving my quality of life |  | O | O | O | O | O |  |
| Improving my sports performance |  | O | O | O | O | O |  |
| Preventing a future illness |  | O | O | O | O | O |  |
| Preventing the expression of a hereditary illness |  | O | O | O | O | O |  |
| Other, please specify ………………………………………………………………………………………………  ………………………………………………………………………………………………………………………… | | | | | | | |

|  | | | | | | | |
| --- | --- | --- | --- | --- | --- | --- | --- |
| **Q17. Please indicate the extent to which you agree or disagree with the following statements:** | | | | | | | |
|  |  | *Completely disagree* | *Disagree* | *Neither disagree/nor agree* | *Agree* | *Completely agree* |  |
| I worry that a personalised diet plan is not effective |  | O | O | O | O | O |  |
| I worry about how my personal data might be used by authorities |  | O | O | O | O | O |  |
| I worry that my personal data may not be treated confidentially |  | O | O | O | O | O |  |
| I worry about how my personal data and test results might be stored |  | O | O | O | O | O |  |
| I worry about how my personal data might be used by personalised nutrition providers |  | O | O | O | O | O |  |
| I worry about how my personal data might be used by advertisers |  | O | O | O | O | O |  |
| I worry about how my personal data might be used by insurance companies |  | O | O | O | O | O |  |
| I worry that my personal data might be accessed by hackers |  | O | O | O | O | O |  |
| Other, please specify………………………………………………………………………………………………... ……..……………………….……………………………….………………………………………………………… | | | | | | | |

|  | | | | | | | |
| --- | --- | --- | --- | --- | --- | --- | --- |
| **Q18. Please indicate to what extent you agree or disagree that the following issues represent barriers to personalised nutrition?** | | | | | | | |
|  |  | *Completely disagree* | *Disagree* | *Neither disagree/nor agree* | *Agree* | *Completely agree* | *Not applicable* |
| Providing different foods for family members |  | O | O | O | O | O | O |
| Difficulties in maintaining healthy eating habits when eating out in restaurants |  | O | O | O | O | O | O |
| Difficulties in maintaining healthy eating habits when eating at other people’s houses |  | O | O | O | O | O | O |
| Difficulties in maintaining diet when travelling |  | O | O | O | O | O | O |
| Difficulties maintaining diet when at work |  | O | O | O | O | O | O |
| Being told to eat foods you don’t like |  | O | O | O | O | O | O |
| Not being recommended to eat foods you like |  | O | O | O | O | O | O |
| My family rejecting the adoption of personalised nutrition |  | O | O | O | O | O | O |
| My friends rejecting the adoption of personalised nutrition |  | O | O | O | O | O | O |
| Society rejecting the adoption of personalised nutrition |  | O | O | O | O | O | O |
| Other, please specify……………………………………………………………………………………………….. ………………………………………………………………………………………………………………………… | | | | | | | |

| **Q19. Please indicate the extent to which the following situations would make you anxious:** | | | | | | | |
| --- | --- | --- | --- | --- | --- | --- | --- |
|  |  | *Not at all anxious* | *Slightly anxious* | *Moderately anxious* | *Very anxious* | *Extremely anxious* |  |
| Taking a blood test |  | O | O | O | O | O |  |
| Providing blood through a finger prick blood test |  | O | O | O | O | O |  |
| Taking a DNA test |  | O | O | O | O | O |  |
|  |  |  |  |  |  |  |  |
| Sending blood samples by mail for analysis |  | O | O | O | O | O |  |
| Sending DNA samples by mail for analysis |  | O | O | O | O | O |  |
| Identifying through a blood test a disease that can’t be treated |  | O | O | O | O | O |  |
| Identifying through a DNA test a disease that can’t be treated |  | O | O | O | O | O |  |
| The security of the blood test data |  | O | O | O | O | O |  |
| The security of the DNA test data |  | O | O | O | O | O |  |
| Other, please specify……………………………………………………………………………………………….. ………………………………………………………………………………………………………………………… | | | | | | | |

|  | | | | | | | |
| --- | --- | --- | --- | --- | --- | --- | --- |
| **Q20. Please indicate the extent to which you would prefer personalised nutrition to be provided through the following communication channels:** | | | | | | | |
|  |  | *Not at all* | *Slightly* | *Moderately* | *Very* | *Extremely* |  |
| Email contact from a named person |  | O | O | O | O | O |  |
| Automated internet service |  | O | O | O | O | O |  |
| Telephone call |  | O | O | O | O | O |  |
| Video call (e.g. Skype) |  | O | O | O | O | O |  |
| Personal meeting |  | O | O | O | O | O |  |
| Apps |  | O | O | O | O | O |  |
| Other, please specify……………………………………..…………………………………………………………. .………………………………………………………………………………………………………………………... | | | | | | | |

| **Q21. Please indicate the extent to which you would prefer the following people or organisations to provide a personalised nutrition service:** | | | | | | | |
| --- | --- | --- | --- | --- | --- | --- | --- |
|  |  | *Not at all* | *Slightly* | *Moderately* | *Very* | *Extremely* |  |
| Family doctor/GP |  | O | O | O | O | O |  |
| Private health organisations |  | O | O | O | O | O |  |
| Dietitian/Nutritionist |  | O | O | O | O | O |  |
| Supermarket |  | O | O | O | O | O |  |
| Other, please specify………………………………………………………………………………………………... ………………………………………………………………………………………………………………………… | | | | | | | |

**Demographics**

**Q22. Please state your occupation:**…………………………………………………………………………

**Q27. Please state the occupation of your partner** (if applicable):……………………………………… …………………………………………….

| **Q23. How healthy do you consider yourself?** | | | | | | | |
| --- | --- | --- | --- | --- | --- | --- | --- |
|  | | | | | | | |
|  |  | *Very unhealthy* | *Unhealthy* | *Moderately unhealthy* | *Healthy* | *Very Healthy* |  |
|  |  | O | O | O | O | O |  |

**Q24. Are you on a restricted diet for a personal reason(s)?** (If yes, please list the personal reason(s) and the foods and drinks you cannot consume as a consequent of that personal reason(s)).

No O

Yes O

Personal reason(s):.................................................................................................................

……………………………………………………………………………………………………….

Foods and drinks that I cannot consume:………………………………………………………..

………………………………………………………………………………………………………..

**Q25. Are you on a restricted diet because of your religion?** (If yes, please list religion and the foods and drinks you cannot consume as a consequent of your religion)

No O

Yes O

Religion:.................................................................................................................................

……………………………………………………………………………………………………….

Foods and drinks that I cannot consume:………………………………………………………..

………………………………………………………………………………………………………..

**Q26. Do you have a food intolerance?**

No O

Yes O

If yes please state which:……………………………………………………………………………….

**Q34. Do you have a food allergy?**

No O

Yes O

If yes please state which:………………………………………………………………………………..

| **Q27. In this box you can post any remarks or comments on this survey you want to share with us.** |
| --- |

**Thank you for your participation!**
