# Supplementary material for: Psychological Determinants of Consumer Acceptance of Personalised Nutrition in 9 European Countries
Source: PLoS One. 2014 Oct 21;9(10):e110614. doi: 10.1371/journal.pone.0110614 (PMC4204923; doi:10.1371/journal.pone.0110614)
Supplement: File S2 — Data File (SPSS format, data relevant to current analysis). (HTM) [file pone.0110614.s002.htm]

 Re: revised PLoS1 


<html><head><meta name="Generator" content="Microsoft Exchange Server">
<!-- converted from text -->
<style><!-- .EmailQuote { margin-left: 1pt; padding-left: 4pt; border-left: #800000 2px solid; } --></style><style>div.PlainText { font-size:120%; font-family:monospace; }</style></head><body>
<font size="2"><span style="font-size:10pt;"><div class="PlainText"><br>
<br>
Hi Lynn and all<br>
<br>
I've read the paper and agree with all the changes. I believe all the&nbsp; <br>
comments have been fully addressed.<br>
<br>
My only suggestion would be to include, when referring to the&nbsp; <br>
Protection Motivation Theory, that the notion of &quot;cost&quot; within that&nbsp; <br>
theory is quite similar to what we call &quot;risk&quot; based on other authors.&nbsp; <br>
Despite the use of italics when referring &quot;costs&quot;, maybe this should&nbsp; <br>
be explicited.<br>
<br>
Please find attached the database with all the variables used.<br>
<br>
Kind regards<br>
rui<br>
<br>
<br>
<br>
<br>
Quoting Lynn Frewer &lt;lynn.frewer@newcastle.ac.uk&gt;:<br>
<br>
&gt; Dear all<br>
&gt;<br>
&gt;<br>
&gt;<br>
&gt; I think this looks OK now..please let me know if you have any&nbsp;&nbsp; <br>
&gt; additional comments by Friday 12.00 otherwise I will assume you are&nbsp;&nbsp; <br>
&gt; all happy with this. Thanks to Barbara for doing most f the&nbsp;&nbsp; <br>
&gt; revisions...I have added a few more. The revisons were pretty minor.<br>
&gt;<br>
&gt; couple of things.<br>
&gt;<br>
&gt; 1.Audrey- do you have a copy of the full questionnaire which we need&nbsp; <br>
&gt;&nbsp; to upload in order to include as a supplementary file.<br>
&gt;<br>
&gt; 2. Rui- we actually need the raw data not the emans&nbsp; ..the idea is&nbsp;&nbsp; <br>
&gt; that, post various publishing scandals, we need to make sure that we&nbsp; <br>
&gt;&nbsp; are transparent and convince people have not made up the results.&nbsp; <br>
&gt; As&nbsp; this is one of the underlying principals of open access&nbsp; <br>
&gt; publishing&nbsp; (and publication of data is normal for RCUK based&nbsp; <br>
&gt; research) could&nbsp; you sent it?We only need the variables use in this&nbsp; <br>
&gt; analysis.<br>
&gt;<br>
&gt;<br>
&gt;<br>
&gt; thanks and best wishes<br>
&gt;<br>
&gt;<br>
&gt;<br>
&gt; Lynn<br>
&gt;<br>
&gt;<br>
&gt;<br>
&gt; Lynn J. Frewer<br>
&gt;<br>
&gt; Professor,&nbsp;&nbsp; Food and Society<br>
&gt; CRE, SAFRD<br>
&gt; Newcastle University<br>
&gt; Newcastle upon Tyne<br>
&gt; NE1 7RU UK<br>
&gt; Tel &#43;44 (0)191 222 8272<br>
&gt;<br>
<br>
<br>
</div></span></font>
</body>
</html>

./help/default.htm?MailPremium

Reply

Reply All

Forward

Re: revised PLoS1

ruipoinhos@fcna.up.pt

To:

M

Lynn Frewer

Cc:

M

Barbara Stewart-Knox ‎[B.Stewart-Knox@bradford.ac.uk]‎‎; 'Fischer, Arnout' ‎[arnout.fischer@wur.nl]‎‎; Sharron Kuznesof‎; Lans, Ivo vander (Ivo.vanderLans@wur.nl)‎; rankin-a10@email.ulster.ac.uk

Attachments:

Consumer acceptance of per~1.sav‎ (335 KB‎)

Categories:

13 August 2014 17:50

|  |  |  |  |
| --- | --- | --- | --- |
|  |  |  |  |
